# Hypothermia does not increase the risk of infection: a case control study

**DOI:** 10.1186/cc10012

**Published:** 2011-02-03

**Authors:** Marlijn Kamps, Laurens LA Bisschops, Johannes G van der Hoeven, Cornelia WE Hoedemaekers

**Affiliations:** 1 Department of Intensive Care, Radboud University Nijmegen Medical Centre PO Box 9101, 6500 HB Nijmegen, The Netherlands

## Abstract

**Introduction:**

Hypothermia may improve outcome in patients after traumatic brain injury, especially when hypothermia is maintained for more than 48 hours. In the acute phase, patients with severe brain injury are more vulnerable to infections. Prolonged hypothermic treatment may further enhance the risk of infection. Selective decontamination of the digestive tract (SDD) reduces the risk of respiratory tract infections. The aim of this study was to investigate the incidence of infections in patients treated with hypothermia and normothermia while receiving SDD.

**Methods:**

In this retrospective case control study 35 patients treated with prolonged hypothermia (cases) were identified and 169 patients with severe brain injury were included (controls). Propensity score matching was performed to correct for differences in baseline characteristics and clinical parameters. Primary outcome was the incidence of infection. The secondary endpoints were the micro-organisms found in the surveillance cultures and infection. In addition, a number of clinical characteristics were assessed.

**Results:**

The demographic and clinical data indicated that the cases and controls were well matched. The overall risk of infection during ICU stay was 20% in the hypothermia groups versus 34.4% in the normothermia group (*P *= 0.388). Pneumonia was diagnosed in 11.4% of patients in both groups (*P *= 1.000). The incidence of meningitis, wound infection, bacteremia, and urinary tract infection was low and comparable between the groups. SDD surveillance cultures indicated a higher colonization with gram-negative bacteria in the rectal samples of the hypothermia patients.

**Conclusions:**

Hypothermia does not increase the risk of infection in patients treated with SDD.

## Introduction

Hypothermia effectively lowers intracranial pressure and may improve neurological outcome and mortality in patients after traumatic brain injury, especially when hypothermia is maintained for more than 48 hours [1-3]. The potential beneficial effect of therapeutic hypothermia is offset by an increased risk of infectious complications. A recent meta-analysis including data from eight high quality trials demonstrated an increased incidence of pneumonia of 51% in patients treated with hypothermia versus 23% in the normothermia group [4]. In addition, pneumonia in hypothermic patients is associated with a more complicated course [5].

Patients with severe brain injury have an increased infection rate varying between 50% and 70% and an increased attributable mortality rate of 5% to 25% [6]. In the first 20 days after injury, the majority of patients with traumatic brain injury die of sepsis or pneumonia [7,8]. It is thought that the post-traumatic immune paralysis is responsible for this increased risk of infection in these patients [9]. Prolonged hypothermic treatment may further enhance the risk of infection. Hypothermia decreases the number of circulating leukocytes as well as their chemotactic and phagocytic capacity [5,10,11]. The release of proinflammatory cytokines such as tumor necrosis factor α and interleukin-1 is diminished by incomplete IkappaB-alpha degradation resulting in reduced NFkappaB-dependent proinflammatory gene expression [12]. Decreased generation of chemokines will diminish the recruitment and activation of neutrophils and other inflammatory cells. In addition, the expression of HSP60 in polymorponuclear leucocytes is lower under hypothermic conditions, thereby reducing the cellular and humoral response against invading microorganisms [13].

Selective decontamination of the digestive tract (SDD) aims at prevention of secondary colonization with potential pathogenic Gram negative bacteria, and yeasts through application of non-absorbable antimicrobial agents in the oropharynx and gastrointestinal tract and systemic administration of cephalosporins during the first four days of admission. SDD reduces the risk of respiratory tract and bloodstream infections and reduces mortality in critically ill patients [14-17]. We hypothesized that the use of SDD in patients treated with mild hypothermia mitigates the earlier mentioned increased risk of infection. The aim of this study was to compare the incidence of infection in patients with severe brain injury treated with prolonged mild therapeutic hypothermia while receiving SDD with normothermic control patients.

## Materials and methods

### Study design, patients and clinical setting

We performed a retrospective case control study to determine the risk of infection in hypothermic and normothermic patients receiving SDD. Cases were patients with severe brain injury who received hypothermic treatment for more than 24 hours. Controls were patients with severe brain injury, who did not receive hypothermic treatment. Both cases and controls were treated with SDD. This study was approved by the local ethical committee of the region Arnhem-Nijmegen. As this was a retrospective analysis of our standard treatment, the ethical committee waived the need for informed consent. The intensive care unit of the Radboud University Nijmegen Medical Centre is a tertiary care ICU with 41 beds. All patients older than 14 years admitted to the ICU between 1 September 2006 and 31 December 2009 with severe brain injury, traumatic or non-traumatic, were analyzed. Patients with severe brain injury were included if they were admitted to the intensive care unit for at least five days and received endotracheal mechanical ventilation. Exclusion criteria were a medical history of an immune deficiency disorder, use of immunosuppressive medication or an age under 15.

### Patient management

All patients were treated according to local protocols and international guidelines. According to our local protocol, cerebral perfusion pressure was maintained at >60 mm Hg. Serum concentrations of sodium, potassium and magnesium were maintained within the normal range. In case of hyperglycemia, patients were treated with continuous insulin infusion therapy aiming at blood glucose levels between 6 and 8 mmol/l. Patients with elevated intracranial pressure were routinely treated with elevation of the head, sedation, and osmotherapy (mannitol and/or hypertonic saline). In case of refractory intracranial hypertension, despite maximal conservative measures, treatment with mild therapeutic hypothermia was considered by the attending physician.

Mild therapeutic hypothermia was induced by rapid infusion of 30 ml/kg bodyweight of cold Ringer's lactate at 4°C followed by external cooling aiming at 32 to 34°C for at least 48 hours. Temperature was measured continuously with a rectal temperature probe (YSI corporated 401, vd Putte Medical, Nieuwegein, The Netherlands). If intracranial pressure normalized, patients were gradually rewarmed to normothermia.

All patients were treated with SDD according to our local protocol based on the study of De Smet *et al. *[17]. Every patient who was admitted to the ICU of our hospital with an expected stay of >48 hours or who was expected to receive mechanical ventilation for >24 hours was treated with SDD. Cefotaxim in a dose of 1,000 mg four times daily was administered intravenously for the first four days. In addition, all patients received topical application of paste in the mouth and a suspension in the stomach which contained polymyxin, tobramycin and amphotericin B. In patients with a tracheostomy the paste was applied around the tracheostomy opening. In patients with a duodenal tube or jejunostomy, the suspension was given both via the gastric tube and the duodenal tube or jejunostomy. Patients with a colostoma or ileostoma received SDD-suppositories twice daily in the distal part of the gut. Surveillance cultures of endotracheal aspirates and oropharyngeal and rectal swabs were obtained at Day 3 after admission and twice weekly thereafter. Based on these surveillance cultures, the SDD regimen was adapted as described by De Smet *et al. *[17]. Patients with a clinical suspicion or documented infection were treated according to standard clinical practice, with the limitation that use of amoxicillin, penicillin, amoxicillin-clavulanic acid, flucloxacillin, piperacillin-tazobactam, meropenem and clindamycin was discouraged. Routine measures to prevent ventilation-associated pneumonia included elevation of the head and aspiration of subglottic secretions.

### Data collection and definitions

Patients were identified using the ICU admission registry. All data were collected from medical charts and laboratory files and were analyzed from the day of admission to the ICU until discharge from the ICU or death.

The primary outcome was the incidence of infection during ICU admission. Pneumonia was defined as a clinical suspicion of pneumonia with positive sputum cultures in the presence of (1) a new infiltrate on the chest X-ray, (2) increased production of purulent sputum, (3) impairment of pulmonary gas exchange not due to left heart failure (two of three criteria were required for the diagnosis). Meningitis was diagnosed in case of any positive culture of the cerebrospinal fluid. Bacteremia was defined as any positive culture of the blood. In the case of coagulase negative staphylococcus, at least two positive blood cultures were required for the diagnosis of bacteremia. A wound infection was diagnosed in the presence of pus combined with disturbed wound healing. A urinary tract infection was defined as the presence of two positive urine cultures with >10^5 ^of the same pathogen or pathogens per ml urine in combination with a clinical suspicion of infection.

The secondary endpoints were to compare the micro-organisms in the surveillance cultures and infection. In addition, a number of clinical characteristics were assessed from the patient files. We defined delayed gastric emptying as the use of parenteral feeding at any time during admission. A patient was considered to be hemodynamically unstable in case of any life threatening rhythm disorder, a mean arterial pressure <50 mmHg or a sudden increase in the need of catecholamines with a least 0.1 ug/kg/minute of norepinephrine or 3.0 ug/kg/minute dobutamine. The minimum and the maximum leukocyte count during ICU stay were determined.

### Statistical analysis

All collected data were analyzed using SPSS 16.0 (SPSS Inc., Chicago, IL, USA). Data are presented as mean with standard deviations (SD) or median with interquartile ranges, unless otherwise indicated. The Students *t*-test, two-tailed Mann-Withney U rank sum test and McNemar test were used to analyze differences between groups. Qualitative data were analyzed using the Chi square test. We used propensity matching in order to ensure that patients and controls were equally balanced on baseline predictors for infection. The propensity score was based on age, gender, body mass index, a history of diabetes, diagnosis at admission, total days of admission, total days of mechanical ventilation, Apache II score, Glasgow coma scale at admission, maximum amount of norepinephrine, dobutamine, midazolam, propofol and insulin infusion, hemodynamic instability, and the occurrence of delayed gastric emptying. Every hypothermic patient was matched to the normothermic patient with the closest propensity score. The Saps II score was not used to compute the propensity score because some patients were under the age of 16 and had an invalid Saps II score. A *P *< 0.05 was considered statistically significant.

## Results

A total of 220 patients with severe brain injury were admitted to the ICU between 1 September 2006 and 31 December 2009. Sixteen patients were excluded because of missing data, none of these patients received hypothermic treatment. Therefore, 204 patients were included in this study. A total of 35 cases were identified that were treated with hypothermia for a median duration of 107 (55 to 168) hours. The remaining 169 patients received normothermic treatment. Hypothermia was initiated in one patient who died from a non-infectious cause nine hours after the start of cooling. This patient was considered a normothermic control. We calculated the propensity scores in all patients. Every hypothermic patient was matched to a normothermic patient with the closest propensity score. Data are presented after propensity score matching.

### Baseline and clinical characteristics

After propensity score matching, baseline variables were comparable between normothermia and hypothermia patients (Table 1). A total of 74.3% of the patients in the normothermia group and 54.2% of the patients in the hypothermia group were male (*P *= 0.167). Although the SAPS II score could not be included in the propensity score, SAPS II scores were comparable between the groups (54.0 (46.8 to 59.5) in the normothermia group versus 54.5 (46.5 to 59.0) in the hypothermia group, *P *= 0.829). Traumatic brain injury and subarachnoid hemorrhage were the most common diagnoses on admission to the ICU with no differences between the groups. The length of stay in the ICU was 11.0 (7.0 to 18.0) days in the normothermia and 10.0 (6.0 to 14.0) days in the hypothermia patients (*P *= 0.830) (Table 2). The number of days on mechanical ventilation was comparable between the groups with 10.0 (7.0 to 17.0) days in the normothermia group and 9.0 (6.0 to 14.0) days in the hypothermia patients (*P *= 0.969). Although hemodynamic instability occurred in only 17.1% of the patients, the majority of patients required cathecholamine infusion to maintain an adequate cerebral perfusion pressure (91.4% and 97.1% of the normothermia and hypothermia patients, *P *= 0.625). More norepinephrine was used in the hypothermia patients compared to the normothermia patients (0.35 (0.16 to 0.55) vs 0.18 (0.13 to 0.26) μg/kg/minute respectively, *P *= 0.053). All patients required sedation, with a significantly higher maximum dosage of midazolam in the hypothermia group compared to the normothermia patients (0.31 ± 0.10 versus 0.25 ± 0.11 mg/kg/hr, *P *= 0.043). Despite matching, significantly more patients received parenteral nutrition in the hypothermia group (68.6% vs 37.1%, *P *= 0.013). A total of 82.9% of the hypothermia treated patients died during ICU admission versus 48.6% of the patients in the normothermia group (*P *= 0.004).

**Table 1:** Demographic data

Demographic data	Normothermia	Hypothermia	*P-*value
**Cases**	35	35	
**Male *n *(%)**	26 (74.3%)	19 (54.2%)	0.167
**Age (yrs)**	42.2 ± 15.6	41.2 ± 14.3	0.754
**BMI (kg/m**^**2**^**)**	25.9 ± 4.7	25.1 ± 2.6	0.387
**Apache II**	24 (21 to 27)	26 (21 to 28)	0.432
**Saps II**	54 (46.8 to 59.5)	54.5 (46.5 to 59.0)	0.829
**Diabetes type II *n *(%)**	1 (2.9%)	0 (0.0%)	NA
**Glasgow coma scale at admission**	7.1 ± 4.0	6.5 ± 3.5	0.460
**Diagnosis on admission *n *(%)**			
**TBI**	19 (54.3%)	20 (57.1%)	1.000
**Multitrauma**	11 (57.9%)	13 (65.0%)	
**Isolated TBI**	8 (42.1%)	7 (35.0%)	
**Subarachnoidal hemorrhage**	12 (34.3%)	8 (22.9%)	0.424
**Subdural hematoma**	1 (2.9%)	1 (2.9%)	1.000
**Intracerebral hemorrhage**	1 (2.9%)	4 (11.4%)	0.375
**Tumor**	2 (5.7%)	2 (5.7%)	1.000

Data are presented as absolute numbers with percentage points, mean ± standard deviation or median with the interquartile range. Yrs, years; BMI, body mass index; Apache II, Acute Physiology and Chronic Health Evaluation II; Saps, Simplified Acute Physiology Score; TBI, Traumatic brain injury; NA, not available.

**Table 2:** Clinical characteristics

	Normothermia (*n *= 35)	Hypothermia (*n *= 35)	*P *value
Length of stay in ICU (days)	11 (7.0 to 18.0)	10.0 (6.0 to 14.0)	0.830
Length of mechanical ventilation (days)	10 (7.0 to 17.0)	9.0 (6.0 to 14.0)	0.969
ICU mortalitity *n* (%)	17 (48.6%)	29 (82.9%)	0.004
Hemodynamic instability *n* (%)	6 (17.1%)	6 (17.1%)	1.000
Use of catecholamines *n* (%)	32 (91.4%)	34 (97.1%)	0.625
Maximum amount of norepinephrine ug/kg/min	0.18 (0.13 to 0.26)	0.35 (0.16 to 0.55)	0.053
Maximum amount of dobutamine ug/kg/min	5.28 (3.33 to 7.19)	3.70 (2.28 to 4.60)	0.086
Patients receiving sedation *n* (%)	35 (100%)	35 (100%)	NA
Maximum amount of propofol mg/kg/hr	3.5 ± 1.5	3.7 ± 1.2	0.737
Maximum amount of midazolam mg/kg/hr	0.25 ± 0.11	0.31 ± 0.10	0.043
Maximum amount of insulin IU/hr	5.0 (3.0 to 7.0)	5.0 (3.0 to 7.0)	0.378
Patients receiving PN *n* (%)	13 (37.1%)	24 (68.6%)	0.013
Leucocytes minimum count 10^^9^/l	7.8 (5.1 to 9.3)	5.8 (4.9 to 8.2)	0.191
Leucocytes maximum count 10^^9^/l	19.4 ± 6.6	20.7 ± 6.4	0.386

Data are presented as absolute numbers with percentage points, mean ± standard deviation or median with the interquartile range. ICU, intensive care unit; NA, not available; PN, parenteral nutrition.

### Incidence of infection

In the hypothermia group, 20.0% of the patients developed an infection during ICU admission compared to 34.3% in the normothermia treated patients (*P *= 0.267) (Table 3). The incidence of pneumonia was comparable between the groups (11.4% in both groups, *P *= 1.0). The incidence of meningitis, bacteremia, wound infection and urinary tract infection was low in both groups.

**Table 3:** Incidence of infections in both groups

	Normothermia (*n *= 35)	Hypothermia (*n *= 35)	*P *value
Patients with an infection *n *(%)	12 (34.3%)	7 (20.0%)	0.267
Pneumonia *n *(%)	4 (11.4%)	4 (11.4%)	1.000
Meningitis *n *(%)	3 (8.6%)	1 (2.9%)	0.625
Bacteremia *n *(%)	3 (8.6%)	2 (5.7%)	1.000
Wound infection *n *(%)	3 (8.6%)	0 (0%)	NA
UTI *n *(%)	0 (0%)	0 (0%)	NA
Total prescribed antibiotics *n *(%)	20 (57.1%)	20 (57.1%)	1.000
Infection with positive culture *n *(%)	11 (31.4%)	7 (20.0%)	0.388

Data are presented as absolute numbers with percentage points. UTI, urinary tract infection

NA, not available.

*Staphylococcus aureus *was most frequently identified as the causative infectious microorganism in both the hypothermia (14.3%) and normothermia (36.3%) group (*P *= 0.375). All *Staphylococcus aureus *strains were meticillin susceptible. The incidence of the other pathogens was relatively low and comparable between the two groups. There were no fungi related infections. Detailed information on micro-organisms found during infection is available in Additional file 1.

### Surveillance cultures

**Figure 1:**
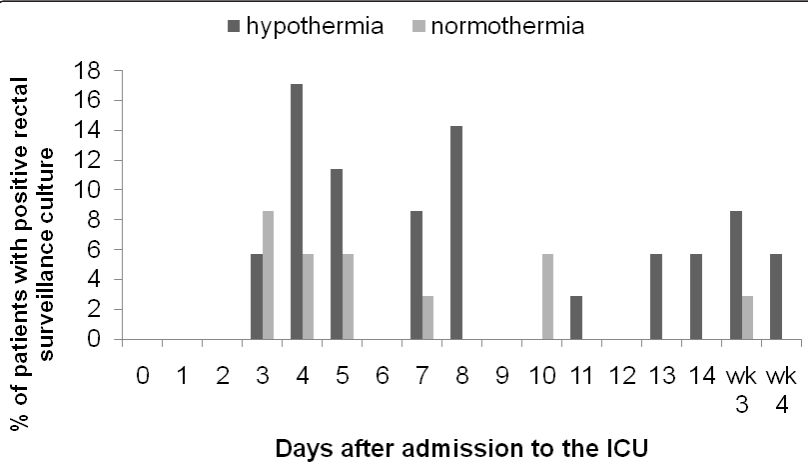
**Rectal colonization in time**. Data are presented as percentage of patients with a positive surveillance culture with Gramnegative bacteria. Wk, week.

**Figure 2:**
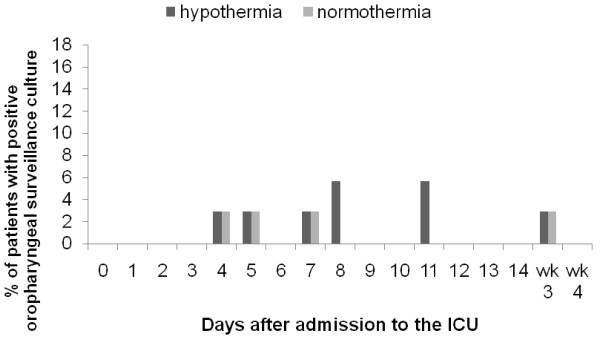
**Oropharyngeal colonization in time**. Data are presented as percentage of patients with a positive surveillance culture with Gramnegative bacteria. Wk, week.

Gram-negative bacteria were isolated from the surveillance cultures in 51.4% of patients treated with hypothermia and 31.4% of patients in the control group (*P *= 0.143) (Table 4). Colonization of the rectum with gram-negative bacteria was significantly more frequent in patients treated with hypothermia compared with normothermia (48.6% versus 20.0% respectively, *P *= 0.041). In contrast, colonization of the upper gastro-intestinal tract and sputum was comparable between the groups with an incidence of 14.3% in the hypothermia patients versus 11.4% in the normothermia patients (*P *= 1.000). No differences were found in the distribution of gram-negative bacteria between the groups. The incidence of *Candida *spp was comparable between the groups with 42.9% in the hypothermia group and 31.4% in the normothermia group (*P *= 0.523). The rate of isolation of gram-negative bacteria from rectal and oropharyngeal swabs was low during ICU stay in both the hypothermia and normothermia patients (Figures 1 and 2). Detailed information on micro-organisms found in the surveillance cultures is available in Additional file 1.

**Table 4:** Positive surveillance culture

	Normothermia (*n *= 35)	Hypothermia (*n *= 35)	*P-*value
**Number of pts with gram negative bacteria in surveillance culture *n *(%)**	9 (25.7%)	18 (51.4%)	0.049
**rectum *n *(%)**	7 (20.0%)	17 (48.6%)	0.041
**oropharynx/sputum *n *(%)**	3 (8.6%)	5 (14.3%)	0.687
**Number of pts with candida spp in surveillance culture *n *(%)**	11(31.4%)	15 (42.9%)	0.523
**rectum *n *(%)**	0 (0%)	0 (0%)	1.000
**oropharynx *n *(%)**	66 (31.4%)	15 (42.9%)	0.523

Data are shown in absolute numbers with percentages.

## Discussion

Under the SDD regime, treatment of patients with mild hypothermia for a prolonged period of time did not increase the risk of infection compared to normothermia patients. Pneumonia and bacteremia were the most frequently diagnosed infections in this population, mostly caused by *Staphylococcus *species. Although the infection risk was comparable between the hypothermia and normothermia group, the colonization rate was significantly higher in the hypothermia compared to the normothermia patients.

This is the first systematic analysis of the effects of SDD in patients with severe brain injury undergoing hypothermia. Although it has been suggested previously that use of SDD in patients with therapeutic hypothermia resulted in low infection rates, detailed information was lacking and no comparison was made with normothermic controls [18]. In patients without SDD, infection rates up to 70% have been reported, with a three-fold increase in the risk of pneumonia in the hypothermia patients [19]. From the present study, it is clear that the risk of (ventilator-induced) pneumonia is low and comparable between hypothermia and normothermia patients.

The incidence of (ventilator induced) pneumonia was comparable between the groups, despite an increased incidence of colonization of the lower digestive tract in hypothermia treated patients. Colonization rates of the oropharynx and stomach with *Staphylococcus aureus *and gram negative bacteria are high in patients after brain injury [20] and may be directly related to the continuous aspiration of pharyngeal or gastric contents. The oropharynx and stomach are independent reservoirs for tracheal colonization with ICU-acquired pathogens and pneumonia [21] and oropharyngeal decontamination appears to be the most effective part of SDD for preventing late-onset pneumonia [22] The relative low incidence of pneumonia in the hypothermia patients despite high colonization rates is most likely related to the preferential colonization of the lower part of the digestive tract. The efficacy of the topical antibiotics in the oropharynx in the present study is comparable in both treatment groups, resulting in similar rates of pneumonia in both groups. The increased colonization rate detected in the rectal samples in hypothermia patients may be explained by the hypothermia induced gastroparesis and bowel dysfunction resulting in inadequate antibiotic concentration in the lower digestive tract.

The rate of isolation of gram-negative bacteria and candida from the surveillance cultures decreases during the course of treatment with SDD [17]. The results of the surveillance cultures in the present study show a lower prevalence throughout the admission period at the ICU. This low prevalence of positive surveillance cultures strongly suggests an effective SDD regime with a high compliance to the protocol.

A major limitation of this study is its retrospective, uncontrolled design, which does not exclude the presence of bias, despite propensity score matching. Patients in the hypothermia group were suffering from intracranial hypertension, refractory to the conservative measures. These patients received higher doses of norepinephrine, sedation and more frequently parenteral nutrition. Mortality in the hypothermia patients was significantly higher compared to the normothermia patients. However, most differences between the groups would increase the risk of infection in the hypothermia patients, thus supporting the hypothesis that SDD mitigates the increased risk of infection during hypothermia.

Infection may have been undiagnosed in the hypothermia group. Serum procalcitonin, C-reactive protein and white blood cell levels are elevated in patients under hypothermia, irrespective of an underlying infection [23]. There is no gold standard for the diagnosis of ventilator-associated pneumonia. Most studies use a combination of clinical, microbiological and radiological criteria. Since fever and white blood cell count could not be used as criteria for the diagnosis, we chose a number of clinical, radiological and microbiological criteria to diagnose pneumonia.

The efficacy and safety of SDD depends on the microbiological setting in which it is used. In settings with high levels of endemic, multidrug resistant gram negative bacteria or methicillin-resistant *Staphylococcus aureus*, SDD is associated with an increased selection of these pathogens [24-26]. In The Netherlands, with a low incidence of multidrug resistant organisms, SDD is not associated with increased selection or induction of antibiotic resistance [27,17]. We used the propensity score matching to correct for differences between the groups. Despite matching, small differences in the use of norepinephrine, midazolam and parenteral nutrition persisted. The retrospective observational nature of this study does not allow us to correct for these differences. Since most of these differences will result in an increased infection risk in the hypothermia treated patients, it is highly unlikely that these differences would considerably affect the conclusions of this study.

## Conclusions

In the setting of a low incidence of multidrug resistant organisms, SDD is a safe method to decrease the risk of infectious complications in patients treated with mild hypothermia for more than 24 hours. Although the results of the surveillance cultures support the hypothesis that oropharyngeal decontamination is the most effective part of the SDD regimen, a randomized controlled clinical trial is needed to establish its exact contribution to the prevention of infectious complications during hypothermia.

## Key messages

• Hypothermia does not increase the risk of infection in patients under SDD.

• Oropharyngeal decontamination may be a more effective part of the SDD regimen, but its exact contribution to the prevention of infections needs to be established.

## Abbreviations

APACHE II: Acute Physiology and Chronic Health Evaluation; BMI: body mass index; HSP 60: heat shock protein 60; ICU: intensive care unit; NA: not available; PN: parenteral nutrition; SAPS: Simplified Acute Physiology Score; SD: standard deviation; SDD: selective decontamination of the digestive tract; SPP: species; TBI: traumatic brain injury; UTI: urinary tract infection; Wk: week.

## Competing interests

The authors declare that they have no competing interests.

## Authors' contributions

MK, CH and LB participated in the design of the study, collected the data and performed the statistical analysis. All authors helped to analyze the results and to draft the manuscript. All authors read and approved the final manuscript.

## Supplementary Material

Additional file 1 **Supplemental tables**. Table S1: Incidence of infections in both groups. Table S2: Positive surveillance cultures. *Staphylococcus.aureus *was most frequently identified as the causative infectious microorganism in both the groups, followed by coagulase negative staphylococci. The incidence of the other pathogens was relatively low and comparable between the two groups. There were no fungi related infections. *Escherichia coli *and *Pseudomonas *spp accounted for most of the gram-negative colonizations. No differences were found in the distribution of gram-negative bacteria between the groups. Click here for file
