# Supplementary material for: Hypothermia does not increase the risk of infection: a case control study
Source: Crit Care. 2011 Feb 3;15(1):R48. doi: 10.1186/cc10012 (PMC3221978; doi:10.1186/cc10012)
Supplement: Additional file 1 — Supplemental tables. Table S1: Incidence of infections in both groups. Table S2: Positive surveillance cultures. Staphylococcus.aureus was most frequently identified as the causative infectious microorganism in both the groups, followed by coagulase negative staphylococci. The incidence of the other pathogens was relatively low and comparable between the two groups. There were no fungi related infections. Escherichia coli and Pseudomonas spp accounted for most of the gram-negative colonizations. No differences were found in the distribution of gram-negative bacteria between the groups. [file cc10012-S1.DOC]

**Additional file 1.**

**Table S1: Incidence of infections in both groups**

|  | **Normothermia**  **(n=35)** | **Hypothermia**  **(n=35)** | **Pvalue** |
| --- | --- | --- | --- |
| **Infection with positive culture n(%)** | 11 (31.4%) | 7 (20.0%) | 0.388 |
| - **Staphylococcus Aureus** | 4 (36.3%) | 1 (14.3%) | 0.375 |
| - **Coagulase negative staphylococci** | 3 (27.3%) | 1(14.3%) | 0.625 |
| - **Streptococcal bacteria** | 1 (9.1%) | 1(14.3%) | 1.000 |
| - **Pseudomonas spp** | 2(18.2%) | 0 (0%) | NA |
| - **Enterococcus spp** | 1 (9.1%) | 1(14.3%) | 1.000 |
| - **Serratia spp** | 0 (0%) | 1(14.3%) | NA |
| - **Enterobacteriacea** | 0 (0%) | 1(14.3%) | NA |
| - **Anaerobic bacteria** | 0 (0%) | 1(14.3%) | NA |
| - **Fungi** | 0 (0%) | 0 (0%) | NA |

Data are presented as absolute numbers with percentage points. NA = not available

**Table S2: Positive surveillance cultures**

Data are shown in absolute numbers with percentages.

|  | **Normothermia**  **(n=35)** | **Hypothermia**  **(n=35)** | **P value** |
| --- | --- | --- | --- |
| **Microorganisms in surveillance culture** |  |  |  |
| - **Escherichia coli n(%)** | 5 (45.5%) | 13 (72.2%) | 0.057 |
| - **Pseudomonas spp n(%)** | 3 (27.3%) | 3 (16.7%) | 1.000 |
| - **Klebsiella spp n(%)** | 2 (18.2%) | 3 (16.7%) | 1.000 |
| - **Enterobacteriaceae n(%)** | 0(0%) | 5 (27.8%) | NA |
| - **Serratia spp n(%)** | 1 (9.1%) | 1 (5.6%) | 1.000 |
| - **Burkholderia cepacia n(%)** | 0 (0%) | 1 (5.6%) | 0.096 |
